# Right Carotid Artery Origin Compression Caused by Endovascular Repair for Kommerell Diverticulum Associated with a Right-Sided Aortic Arch

**DOI:** 10.3400/avd.cr.24-00002

**Published:** 2024-04-10

**Authors:** Hiroomi Nishio, Atsushi Iwakura, Naoki Takahashi, Kenji Aida, Kyozo Inoue, Fumie Takai, Masanosuke Ishigami, Hiroshi Yukawa, Hisashi Sakaguchi, Takashi Fukushima, Fujio Hayashi

**Affiliations:** 1 Department of Cardiovascular Surgery, Osaka Red Cross Hospital, Osaka, Osaka, Japan; 2 Department of Cardiology, Osaka Red Cross Hospital, Osaka, Osaka, Japan; 3 Department of Cardiovascular Surgery, Yodogawa Christian Hospital, Osaka, Osaka, Japan

**Keywords:** Kommerell diverticulum, carotid artery, right-sided arch

## Abstract

Owing to the unique anatomical features, the endovascular repair for Kommerell diverticulum poses a surgical challenge. An 80-year-old, asymptomatic female with Kommerell diverticulum and associated right-sided aortic arch underwent an endovascular repair, consisting of an aortic arch endografting with a proximal extension, axillo-axillary crossover bypass, and right subclavian parallel endografting. An additional stent was promptly placed retrogradely at the right carotid artery origin as the completion aortography revealed an ostial occlusion. During the 6th month follow-up, she remained well without any neurological deficits. This report elucidated the disease-specific and procedure-related causes leading to right carotid artery ostium occlusion.

## Introduction

A Kommerell diverticulum is a remnant of the primitive aortic arch that failed to regress.^1)^ Historically, this rare aortic pathology is treated via open surgery, including aberrant subclavian artery resection and ligation with a subclavian to carotid bypass or transposition to alleviate compressive symptoms. Recent advances in treatment modality enabled a growing number of patients with Kommerell diverticulum to be managed endovascularly. A previous study reported a specific tendency for an endovascular stent graft placement for elderly or asymptomatic patients.^2)^ However, several impediments may hinder the endovascular repair of Kommerell diverticulum, including the access artery size, distal aortic arch tortuosity, and proximal neck morphology.^3^^,^^4)^ Additionally, a steep aortic arch angulation and difficulty achieving proximal extension of the endograft are also among these obstacles. In this case report, we describe yet another anatomical obstacle to the endovascular repair of Kommerell diverticulum associated with a right-sided aortic arch.

## Case Report

**Figure 1:**
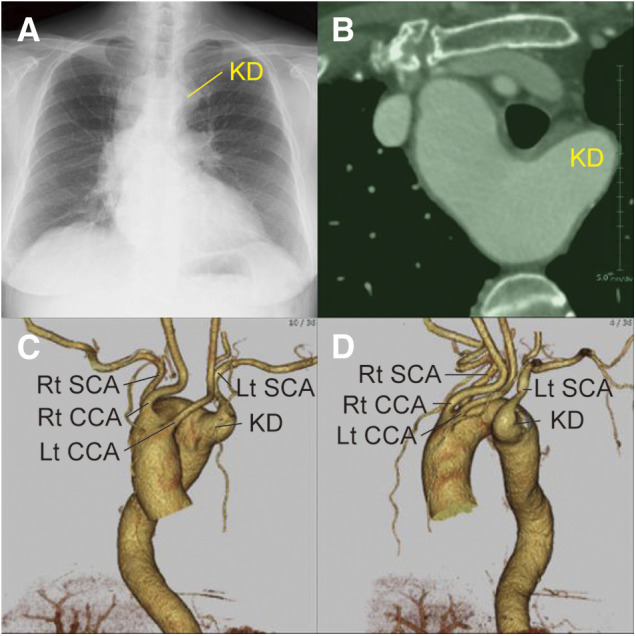
Fig. 1 Preoperative imaging studies. (**A**) Chest X-ray. (**B–D**) CT images. CT: computed tomography; KD: Kommerell diverticulum; Lt CCA: left common carotid artery; Lt SCA: left subclavian artery; Rt CCA: right common carotid artery; Rt SCA: right subclavian artery

An 80-year-old, asymptomatic woman with a history of hypertension and hyperlipidemia was referred to our institution for further evaluation and treatment of Kommerell diverticulum associated with a right-sided aortic arch, which was identified during a regular health checkup as a chest radiograph abnormality (**Fig. 1A**). Computed tomography (CT) revealed an aberrant left subclavian artery posterior to the trachea and esophagus and a 21-mm-diameter Kommerell diverticulum, with a 57-mm distance to the opposite aortic wall (**Figs. 1B**–**1D**). Surgical intervention was anticipated to prevent rupture and dissection. Owing to the advanced age and a lack of compressive symptoms, an endovascular repair was proposed to the patient. The aortic arch was steeply angulated, and the distance between the aortic arch angle and the proximal edge of the Kommerell diverticulum was rendered insufficient as a landing zone. Thus, extended endovascular placement of two stent grafts was planned over the aortic arch, from the origin of the right subclavian artery to the descending aorta distally to the Kommerell diverticulum, with an axillo-axillary crossover bypass and right subclavian parallel endografting.

**Figure 2:**
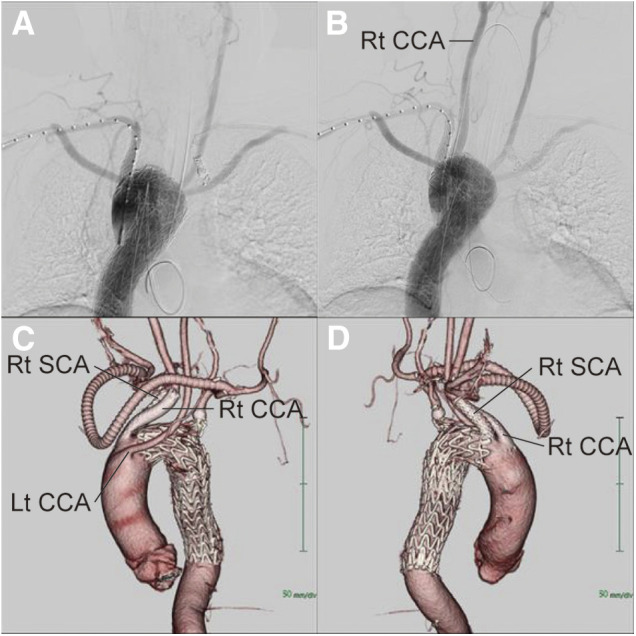
Fig. 2 Intraoperative aortography and postoperative CT. Intraoperative aortography before (**A**) and after (**B**) an additional stent placement at the right common carotid artery ostium. (**C** and **D**) Three-dimensional CT images prior to discharge. CT: computed tomography; Lt CCA: left common carotid artery; Rt CCA: right common carotid artery; Rt SCA: right subclavian artery

Surgery was performed, where an axillo-axillary crossover bypass was constructed using a ringed expanded polytetrafluoroethylene graft. A 20-F introducer sheath was then percutaneously inserted through the right common femoral artery. A 6-F balloon guide catheter (OPTIMO PPI SHEATHLESS KIT; Tokai Medical Products, Inc., Kasugai, Japan) was inserted through the left brachial artery to prevent left vertebral artery embolization during aortic stent graft placement. A 34 × 100-mm stent graft (GORE TAG Conformable Thoracic Stent Graft with ACTIVE CONTROL System; W.L. Gore & Associates, Inc., Flagstaff, AZ, USA) with an oversize ratio of 110% was deployed in the distal aortic arch using an imaging catheter inserted from the right brachial artery to cover the Kommerell diverticulum origin. More proximally, a 37 × 100-mm stent graft (GORE TAG Conformable Thoracic Stent Graft with ACTIVE CONTROL System) with an oversize ratio of 120% was deployed to cover the right subclavian artery origin. An 8 × 59-mm balloon-expandable stent graft (GORE VIABAHN VBX Balloon Expandable Endoprosthesis; W.L. Gore & Associates, Inc.) was introduced via the right brachial artery and positioned at the right subclavian artery origin to protrude into the aortic arch. Concurrent inflation of the endografts placed in the aortic arch and right subclavian artery was performed. The aberrant left subclavian artery was embolized just proximal to the left vertebral artery origin using a plug and coils. An occlusion at the origin of the right carotid artery was shown via aortography (**Fig. 2A**). Thereafter, the right common carotid artery was percutaneously approached at the cervical site, and a guide wire was easily advanced into the ascending aorta. Contrast media injection from a sheath placed in the right carotid artery revealed an antegrade blood flow and stenosis at the origin of the right common carotid artery. Hence, an additional 10 × 60-mm self-expandable stent (S.M.A.R.T. CONTROL Vascular Stent System; Cordis US Corporation, Miami Lakes, FL, USA) was placed at the ostium of the right common carotid artery. Completion aortography revealed patency of the right carotid artery and exclusion of the diverticulum (**Fig. 2B**). The patient’s postoperative course was uneventful. Postoperative CT revealed that the proximal edge of the stent graft placed in the aortic arch did not reach the ostium of the right carotid artery (**Figs. 2C** and **2D**), and a small type Ia endoleak was observed. The patient was doing well at the 6-month regular checkup.

**Figure 3:**
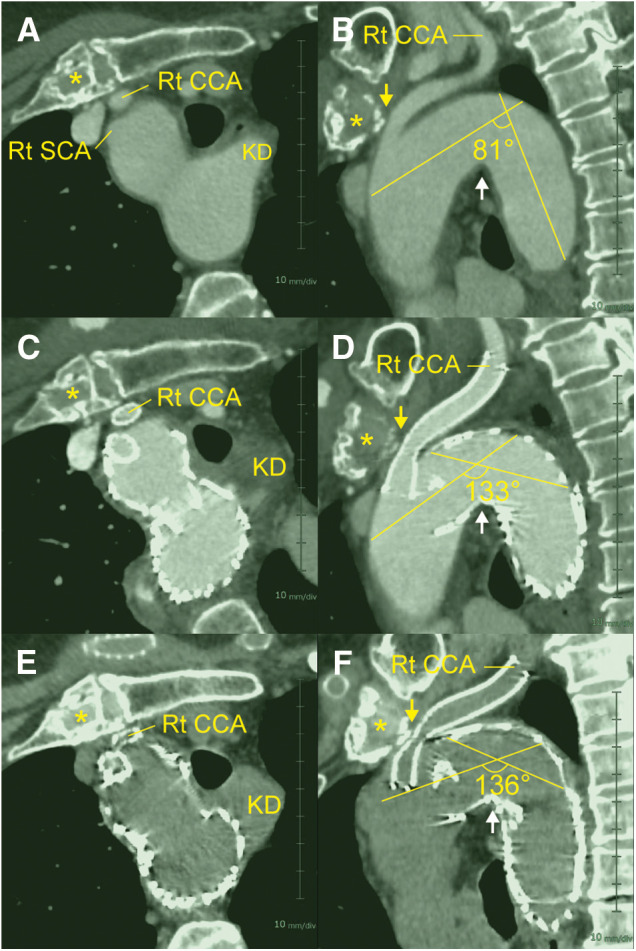
Fig. 3 Morphological changes in the right common carotid artery and aortic arch. The CT scans were taken preoperatively (**A** and **B**), prior to discharge (**C** and **D**), and 6 months postoperatively (**E** and **F**). The most compressed site of the right common carotid artery at each time point was demonstrated axially or sagittally. Lengths between the first right rib (yellow arrow) and the inflection point of the lesser curvature of the aortic arch (white arrow), measured in planes passing parallel to the proximal portion of the right common carotid artery via the protuberant part of the first right rib (white arrow), preoperatively, prior to discharge, and 6 months postoperatively, were 40.0 mm, 35.2 mm, and 35.8 mm, respectively. The angle of the aortic arch was measured using center lines. KD: Kommerell diverticulum; Rt CCA: right common carotid artery; Rt SCA: right subclavian artery

An anatomical CT scan analysis revealed that the lengths between the first right rib and the inflection point of the lesser curvature of the aortic arch measured at each time point of preoperative, postoperative, and 6-month follow-up after the operation were 40.0 mm, 35.2 mm, and 35.8 mm, respectively (**Fig. 3**). Furthermore, the aortic arch angles were 81°, 133°, and 136°, respectively. CT imaging at the 6-month follow-up revealed a patent right carotid artery despite its markedly deformed origin. Additionally, the aortic arch became less angulated, indicating that a spring-back force of the endograft anteriorly dislocated the aortic arch.

## Discussion

In this case report, a patient with Kommerell diverticulum and associated right-sided aortic arch underwent thoracic endovascular aortic repair, and the origin of the right carotid artery was occluded due to an external compression. This case highlighted two important concerns regarding thoracic endovascular aortic repair in patients with a right-sided aortic arch. The thorax and aortic arch intrinsically compress the right carotid artery origin. The anterior displacement of the aortic arch caused by a spring-back force of endografts can render the origin of the right carotid artery narrower.

First, the thorax and aortic arch intrinsically compress the right carotid artery origin in patients with a right-sided aortic arch. The right common carotid artery was slightly compressed by the sternocostal joint and aortic arch in the preoperative imaging of this patient (**Figs. 3A** and **3B**). Thus, full expansion was not achieved even after stent placement, which also indicates presence of an external compression by the surrounding tissues. As Tola et al. have first reported a case with a right-sided aortic arch and a severe narrowing at the origin of the right carotid artery, which was positionally occluded preoperatively,^5)^ the right carotid artery associated with a right-sided aortic arch is susceptible to stenosis.

Second, the anterior displacement of the aortic arch caused by a spring-back force of endografts can render the origin of the right carotid artery narrower when a right-sided aortic arch-involving pathology is endovascularly treated. In the present case, the right carotid artery and aortic arch ran parallel to each other and were aligned with the first rib and vertebra (**Fig. 3**). Moreover, minimal space was observed around the aortic arch anteriorly and posteriorly. Consequently, the aortic arch was anteriorly displaced once the endografts were deployed in the arch. In **Fig. 3**, no alterations were observed 6 months postoperatively in the length between the tip of the rib and the inflection point of the lesser curvature of the arch compared to that observed at discharge. However, the arch became less angulated, indicating that the right carotid artery compression was mainly attributed to the spring-back force of the endografts. As previously reported,^6^^,^^7)^ a spring-back force features a tendency to return to a straight position and affects more at both ends of an endograft, sometimes causing a stent graft-induced new entry and an endoleak. Moreover, this case showed that an external right carotid artery compression is yet another complication caused by the spring-back force of an endograft. A thorough preoperative review of the positional relationship between the right carotid artery, aortic arch, thorax, and vertebra would be beneficial in predicting an occlusion of the right carotid artery ostium.

However, it remains unclear which revascularization strategy would be appropriate for treating patients endovascularly with a right-sided aortic arch where an aortic endograft needs to cover the origin of the right subclavian artery. Since Okada et al. first reported a case with a Kommerell diverticulum who was managed endovascularly,^3)^ a number of reports describing an endovascular repair for the pathology with or without a left subclavian revascularization were published in the early days. In 2015, two groups adopted the right subclavian revascularization technique, a carotid to subclavian bypass or a parallel endografting, to extend an aortic endograft beyond the steep aortic arch angle.^8^^,^^9)^ To date, more than ten patients with a right-sided aortic arch managed endovascularly without sternotomy or thoracotomy were reported to cover the origin of the right subclavian artery. For the majority of these cases, the right subclavian artery bypass was constructed. Notably, Zalle et al. reported a similar case with a right-sided arch who showed compression of the right carotid artery origin after arch endografting, which was relieved through bare stent placement.^10)^ Although a prompt stent placement of the right carotid artery ostium should be considered as a rescue treatment, a spring-back force of the endograft persistently affects the origin of the right carotid artery afterward, as was observed in the present case. Thus, if an external compression of the right carotid artery after arch endografting is anticipated preoperatively, a parallel endografting may be appropriate as a right subclavian artery revascularization because the right subclavian artery would work as an inflow of a secondary right carotid artery bypass grafting.

## Conclusion

Herein, we report a patient with Kommerell diverticulum and a right-sided aortic arch who underwent endovascular repair through a proximal aortic arch endograft extension to the right subclavian artery origin. However, the disease-specific anatomy and a spring-back force of the endograft caused this technique to result in occlusion at the origin of the right carotid artery. This occlusion was promptly relieved by an additional stent placement. A thorough preoperative review of the positional relationship around the right carotid artery would help predict an ostial occlusion.

## Acknowledgments

The authors would like to thank Enago (www.enago.jp) for the English language review.

## Informed Consent

The local ethics committee approved this case study on January 11, 2024 (reference number: J-0555), and informed consent was obtained from the patient.

## Author Contributions

Study conception: HN, AI, NT, KA, and KI

Data collection: HN, NT, KA, KI, HS, and TF

Analysis: HN, NT, KA, KI, and FH

Investigation: HN, NT, KA, KI, HY, FT, and MI

Manuscript preparation: HN and AI

Funding acquisition: none

Critical review and revision: all authors

Final approval of the article: all authors

Accountability for all aspects of the work: all authors

## Disclosure Statement

All authors declare no conflict of interest.
